# “Female Preponderance” of Depression in Non-clinical Populations: A Meta-Analytic Study

**DOI:** 10.3389/fpsyg.2016.01398

**Published:** 2016-09-15

**Authors:** Kui Wang, Han Lu, Eric F. C. Cheung, David L. Neumann, David H. K. Shum, Raymond C. K. Chan

**Affiliations:** ^1^Neuropsychology and Applied Cognitive Neuroscience Laboratory, CAS Key Laboratory of Mental Health, Institute of PsychologyBeijing, China; ^2^Department of Psychology, College of Humanities and Social Sciences, University of Chinese Academy of SciencesBeijing, China; ^3^Joint Master in Neuroscience, University of StrasbourgStrasbourg, France; ^4^School of Psychology, Beijing Normal UniversityBeijing, China; ^5^Castle Peak Hospital, Hong Kong Special Administrative RegionHong Kong, China; ^6^Menzies Health Institute Queensland and School of Applied Psychology, Griffith UniversityGold Coast, QLD, Australia

**Keywords:** gender difference, depression, age, social gender role, economic status, BDI

## Abstract

Clinical observations and research suggest a female preponderance in major depressive disorder. However, it is unclear whether a similar gender difference is found for the reporting of depressive symptoms in non-clinical populations. The present meta-analysis was conducted to address this issue. We searched for published papers targeting non-clinical populations in which the 21-item Beck Depression Inventory (BDI) was used. Eighty-four papers (91 studies) published between 1977 and 2014 were included in the final meta-analysis, which comprised 23,579 males and 29,470 females. Females in the general population reported higher level of depressive symptoms than males (*d* = -0.187, corresponding to 1.159 points in the 21-item BDI). This pattern was not found to influence by years of publication, socioeconomic status, or version of the BDI used. Using age group as a moderator, studies with adolescents and young adults were found to show a smaller effect size than studies with older participants. Our results appear to confirm the “female preponderance” in the level of self-report depressive symptoms in the general population, and support the social gender role theory in explaining gender difference over biological susceptibility theory and evolutionary theory.

## Introduction

While results of several large-scale studies seem to suggest a higher level of depressive symptoms in females than males in the general population (Nolen-Hoeksema et al., 1999; Koivumaa-Honkanen et al., 2004; Aalto et al., 2012; Lopez Molina et al., 2014), consistent results are not always reported (Hammen and Padesky, 1977; Kim et al., 2011). However, due to the apparent agreement of this idea with the widely accepted “female preponderance” of major depressive disorder (Weissman and Klerman, 1977), this notion has been widely accepted, despite the lack of any systematic investigation in the general population.

Three representative theories purport to explain this gender difference in the level of depressive symptoms in the general population. First, the social gender role theory is based on females’ long-standing disadvantaged social status which exists across many different cultures (Gove, 1977; Mirowsky, 1996). The theory states that lower status in the workplace and in heterosexual relationships cause chronic strain, decrease the sense of mastery and stimulate the use of a rumination strategy for emotional coping, thus contributing to a higher vulnerability to depression in females. Second, the biological susceptibility theory is based on the observation that clinical depression tends to be associated with events in the female reproductive cycle. Thus, the effect of female hormones has been proposed as a potential cause for the observed gender difference in depression (Janowsky and Rausch, 1985). Third, evolutionary psychologists have noted that males tend to value physical appearance more than females when choosing a partner. In modern societies, the mass media has repeatedly promoted the images of idealized attractive females and these images are readily accepted by both males and females. Thus, females may become increasingly dissatisfied with their own physical appearances due to increased pressure for mate selection, and as a result are more likely to become depressed when their appearances fall short of the ideal (Buss, 2003). All of these theories have been elaborated extensively (Weissman et al., 1993), but a direct comparison between them has not been carried out.

Unlike the ratio of gender difference in patients with diagnosed major depressive disorder (about 2:1), which is supported by many community surveys, epidemiological studies, clinical observations, and meta-analyses (Weissman and Klerman, 1977; Boyd and Weissman, 1981; Ayuso-Mateos et al., 2001; Hankin and Abramson, 2001; Seedat et al., 2009; Luppa et al., 2012), empirical support for the difference in level of depressive symptoms in the general population is less clear (Hill and Needham, 2013). In fact, some large scale studies have not found any gender difference in the level of depressive symptoms in non-clinical samples (Hammen and Padesky, 1977; Kim et al., 2011). Depressive symptoms are frequently measured by self-report measures, such as the Beck Depression Inventory (BDI; Beck et al., 1961). By combining results from different studies, a meta-analysis can answer not only whether there is a gender difference in the level of depressive symptoms in non-clinical populations, but also assess the magnitude of this difference. In view of the widely observed gender difference in diagnosed major depressive disorder (Weissman and Klerman, 1977; Boyd and Weissman, 1981; Blazer et al., 1998; Hankin et al., 1998; Ayuso-Mateos et al., 2001; Seedat et al., 2009; Luppa et al., 2012) and the continual nature of depression, we expected to find a gender difference in the level of depressive symptoms in non-clinical populations as well.

Using moderator analyses, it is also possible to examine what factors might be related to gender difference in the level of depressive symptoms. For example, the association between depression and economic status has been well-established (McGrath et al., 1990; Steptoe et al., 2007; Kessler et al., 2009). In addition to a stable ratio of gender difference in major depressive disorder (especially in industrialized countries), people living in poverty have been found to show a higher prevalence of diagnosed depression than those of a higher economic status in the same country (Kessler et al., 2009). However, whether gender difference in the level of depressive symptoms in the general population, if it exists, is influenced by socioeconomic status is largely unknown. Similarly, epidemiological surveys and family studies suggest that the rate of depression has been increasing over the past few decades (Angst, 1985; Klerman and Weissman, 1989; Cross-National Collaborative Group, 1992; Weissman et al., 1993), which may also influence gender difference in the level of depressive symptoms. For example, with epidemiological survey data collected in the United States, Canada, Germany, and New Zealand, Weissman et al. (1993) suggested that gender difference in the rate of depression is decreasing due to the increased prevalence of depression in men. This question has not been addressed in the general population. By including year of publication and socioeconomic status of countries/regions where the study was carried out as moderators, a meta-analysis could address both of the above questions. In addition, taking year of publication as a potential moderator offers us an opportunity to observe whether the expected gender difference in the general population is decreasing or not, since such a trend has been suggested in diagnosed depression (Klerman and Weissman, 1989).

Finally and theoretically, taking participants’ age as a moderator in the meta-analysis offers the opportunity to test which of the three theories mentioned above best explain gender difference in the level of depressive symptoms in the general population. If the social gender role theory is valid, we would expect gender difference in the level of depressive symptoms to increase with gender socialization processes, and to remain unchanged when an individual attains the socially accepted gender role. If female hormones best explain this phenomenon, we would expect gender difference in the level of depressive symptoms to decrease in the age period when the production and release of female hormones decrease (Mirowsky, 1996), especially in the elderly group. Finally, if gender difference in the level of depressive symptoms was mainly due to increased dissatisfaction of physical appearance and increased mate selection pressure, we would expect the largest gender difference to be found in young adulthood when an individual’s prime concern is to develop intimacy and find a mate (Erikson, 1975).

In summary, the primary aim of the present research was to conduct a meta-analysis to investigate whether there is gender difference in the level of depressive symptoms in the general population and to examine how this gender difference, if it exists, varies with time and socioeconomic status of a country/region. Finally, we aimed to examine which of the three aforementioned theories best explains this gender difference, if it exists, by including age as a moderator. The findings of the current meta-analysis may contribute to our understanding of clinical depression, since depression has been suggested as a spectrum ranging from subthreshold depressive symptoms to clinically diagnosed depression (Seligman, 1978; Flett et al., 1997; Bowins, 2015).

## Materials and Methods

### Literature Search

First developed in 1961, the Beck Depression Inventory (Beck et al., 1961) is one of the best known and most widely used instruments for assessing the presence and severity of depressive symptoms in individuals aged 13 years and above (Beck et al., 1996). It is considered a gold standard of self-rating scales of depressive symptoms (Cusin et al., 2010). Good agreement between the BDI and a clinical diagnosis of depression has been repeatedly demonstrated (Beck et al., 1996; Schotte et al., 1997; Lasa et al., 2000; Gomes-Oliveira et al., 2012; Araya et al., 2013). The cross-cultural acceptance of the BDI also makes it a good tool to use in studying the underlying construct of depression in different cultural settings. The BDI has several versions, with the 21-item version most frequently adopted in either English or non-English languages. Thus, in the present meta-analysis, we only selected studies that had used the 21-item BDI for a better cross-cultural comparison of depressive symptoms.

Potential journal articles were identified from PubMed, EBSCO (PsychINFO, PsychARTICLE), and Web of Knowledge between 1961 and February 2014. The keywords used were ‘Beck Depression Inventory’ or “BDI” in the text but not “patient” in either the title or the abstract of a paper. The search yielded an initial pool of 2174 articles.

**FIGURE 1:**
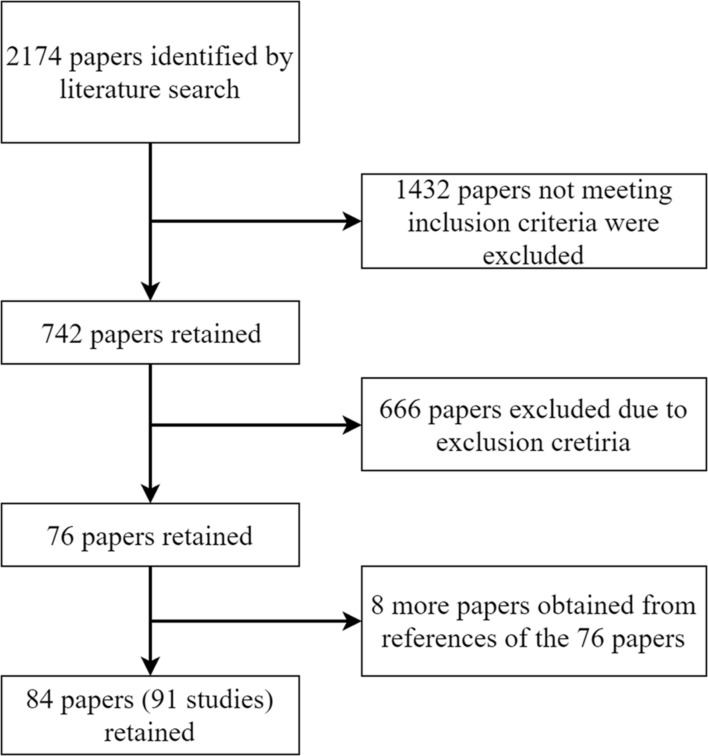
**Flowchart for the inclusion of published data for the current meta-analysis**.

The 2174 papers were checked against criteria for inclusion and exclusion (**Figure 1**).

The inclusion criteria were:

(1) Published in English (106 studies excluded);
(2) Participants included both genders (507 studies excluded);
(3) Participants were selected from the normal population (e.g., not elite athletes, transsexuals, cancer survivors, or cross-culturally married couples), and were free from physical and mental disorders (e.g., eating disorder, depression), addiction of any kind or obvious stress (e.g., care givers, survivors of accidents, victims of sexual assault, or recently bereaved; 800 studies excluded);
(4) The 21-item BDI was used (19 studies excluded).

After this step, a total of 742 papers remained and they were then subjected to the following six exclusion criteria:

(1) Studies that included data already reported in a previous study (one study excluded);
(2) Data for meta-analysis (means and SDs for both genders or exact *t* values or *F* values for gender means comparison) were not available (651 studies excluded);
(3) The BDI was used to measure the effect of a manipulation (10 studies excluded);
(4) Participants were asked to respond to the BDI under faked conditions (one study excluded);
(5) Not all 21 items of the BDI were used in the study (one study excluded);
(6) Case study (two studies excluded).

After this step, 76 papers were retained. A review of the reference list of these 76 papers generated another eight additional papers which were included in the meta-analysis. Consequently, the final sample included 84 papers, seven of which had two participant groups. Thus, the final meta-analysis contained 91 studies.

The socioeconomic status of a country/region where the study was carried out was measured using two indices: the gross domestic product (GDP) per capita and the Gini index. GDP data for 2013 (the most recent available data) were retrieved from the World Bank online database^1^. The Gini index reflects the income distribution of a nation’s residents, and is the most commonly used measure of inequality (Gini, 1909). Since the Gini index data for each country/region provided by the World Bank contained many missing data, we retrieved the relevant data from Quandl^2^, a search engine with financial, economic, and social studies. Nevertheless, the Gini indices for four studies were not available (one from the United Arab Emirates, one from Bahrain and two from Saudi Arabia). We then compared the Gini indices of regions/countries included in the present analysis with data available from the World Bank and found that the data tallied well.

It has been suggested that different versions of the BDI used across studies may be the cause of inconsistent findings in gender difference in the level of depressive symptoms in the general population (O’Hara et al., 1998). For this reason, we also investigated whether gender difference in the level of depressive symptoms may be influenced by the BDI version used (English vs. non-English; for English version: BDI-I vs. BDI-II). This measure was taken as a potential moderator for analysis. Overall, potential moderators in this meta-analysis included the age range of the participants, year of publication, the BDI version used, the GDP per capita, and the Gini index of the country/region where the study was carried out.

### Meta-Analysis Procedure

The present meta-analysis was carried out using the Comprehensive Meta-Analysis (version 2.0) software package (Borenstein et al., 2005). Since we only included studies using the 21-item BDI, the mean difference (the mean BDI score in the male sample minus the mean BDI score in the female sample) was also available as an effect size, together with the widely used Cohen’s *d*. For both measures, a negative difference score indicates a higher BDI depression score in females than in males. The random model was used for calculating the effect sizes due to expected heterogeneity. A funnel plot was used to illustrate potential publication bias and quality of individual studies. In addition, the fail-safe-N, the number of additional unpublished studies with negative effect that would be needed to increase the *p* value for the meta-analysis to above 0.05, was also calculated to estimate publication bias (Rosenberg, 2005).

## Results

### Feature of Studies

The 91 studies were published between 1977 and 2014, with 38 published before 2000 and 53 published in 2000 or later. These studies involved 23,579 males and 29,470 females in 22 different countries/regions from six continents (1 in Africa, 11 in Asia, 25 in Europe, 40 in North America, 3 in Oceania, and 11 in South American). The socio-economic status of these countries varies substantially, with the GDP per capita ranging from USD 4838.50–67458.40 and the Gini index from 27.79 to 63.14. In relation to the versions of the BDI used, 47 studies used the English version of the BDI, among which 22 clearly stated using the BDI-I and 13 stated using the BDI-II. The other 44 studies were carried out with a non-English version of the BDI. Based on the age range of the participants and the sampling approach used, the studies were classified into five groups, adolescents (13 studies), young adults (exclusively university students, 45 studies), middle-aged adults (30–50 year old, five studies), older adults (six studies), and general population aged above 13 years (15 studies). The remaining seven studies could not be classified into any of these groups due to insufficient information. The mean depression score in 86 studies (five did not report the mean total scores, but the *t* or *F* value for between-gender comparison on BDI total score were available for meta-analysis) ranged from 2.08 to 19.30 [five reported a BDI mean total score higher than 13, the cut-off for mild depression on BDI-II (Beck et al., 1988)] for males, and 2.86–19.00 (eight reported a BDI mean total score higher than 13) for females.

### General Analysis

The effect size for gender difference of each study varied from -1.146 to 0.227. Taken together, a random effect size of -0.187 (*SE* = 0.017, 95% *CI* = [-0.220, -0.155]) was found. The small but significant (*Z* = -11.70, *p* < 0.001) effect size indicated that on average females scored about 1.159 points (*SE* = 0.112, 95% *CI* = [-1.379, -0.939]) higher on the 21-item BDI than males. Given the large number of studies, it could be expected that the 91 studies were not homogeneous [*Q*(90) = 223.218, *p* < 0.001, *I*^2^ = 59.681]. Closer inspection showed that the study reported by Goodrich and Weaver (1998) generated a very large effect size of -1.146 (the second largest was -0.576). The sample included only 11 men and 13 women. Removing this study from the pool had a negligible effect on the mean effect size (Cohen’s *d* = -0.186). This study was therefore excluded from further analysis.

**FIGURE 2:**
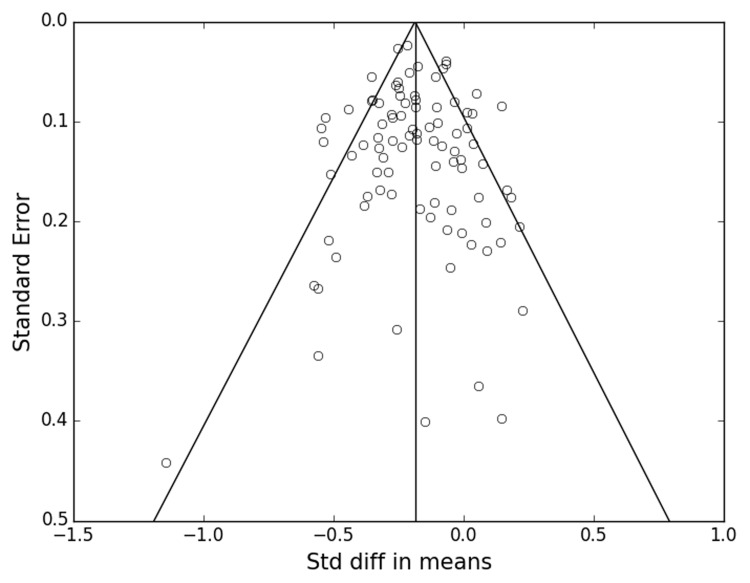
**Funnel plot of standard errors as a function of standard difference in the means**.

Publication bias was evaluated by the fail-safe-*N* and funnel plot. The fail safe N was 6987. **Figure 2** shows the funnel plot with each circle representing an individual study included in the meta-analysis. The circles were generally symmetrical in their distribution, indicating the absence of publication bias. Additionally, most of the circles were located near the top of the funnel, indicating a relatively small standard error, suggesting good methodological quality of these studies.

### Potential Moderator Analysis

Taking age as a moderator, we compared the effect sizes of the five groups of studies, namely adolescents, young adults, middle-aged adults, general population, and older adults. Significant effect sizes (**Table 1**) were generated from each group. Studies in the older adult group, the general population group, and the middle-aged adult group were homogenous. Additionally, the effect sizes generated from these three groups were not significantly different [*Q*(2) = 4.089, *p* = 0.13]. Studies in the adolescent group and the young adult group were heterogeneous, and generated relatively small but significant effect sizes (adolescents: *d* = -0.188; early adults: *d* = -0.157). To test whether the effect sizes of the two younger groups were smaller than those of the three older groups, we created a “young” group including studies with adolescents and young adults, and an “old” group with studies involving middle-aged adults, older adults, and the general population. The 58 studies in the “young” group generated a small but significant effect size of -0.166 (*SE* = 0.024, 95% *CI* = [-0.212, -0.119], *Z* = -11.66, *p* < 0.01), which corresponded to -1.063 (*SE* = 0.167, 95% *CI* = [-1.390, -0.736]) points in the total BDI score. The fail-safe N was 1804. The 26 papers in the “old” group generated an effect size of -0.224 (*SE* = 0.019, 95% *CI* = [-0.262, -0.187], *Z* = -11.76, *p* < 0.01), which corresponded to -1.33 (*SE* = 0.117, 95% *CI* = [-1.562, -1.103]) points in the total BDI score. The fail-safe N was 1283. The *Q*-test indicated that the effect sizes in the “old” group were larger than those in the “young” group [*Q*(1) = 3.702, *p* = 0.05]. Studies that recruited from the general population contained individuals belonging to both the “old” and “young” groups. For this reason, we excluded these studies from the “old” group and found a similar group difference [*Q*(1) = 5.270, *p* < 0.05].

**Table 1:** Effect sizes of gender difference in BDI (Beck Depression Inventory) total score across age groups.

Age	K	NM	NF	Cohen’s *d*	*SE*	95% CI	Z	Heterogeneity		Fail-safe-N
								*Q*	*I*^2^	
Adolescents	13	4640	4852	-0.188	0.042	-0.271, -0.105	-4.44^∗∗^	39.745^∗∗^	69.808	199
Early adults	45	6599	9389	-0.157	0.029	-0.214, -0.100	-5.37^∗∗^	109.94^∗∗^	59.978	773
Middle adults	5	3497	4966	-0.251	0.052	-0.352, -0.149	-4.83^∗^	5.567	28.150	66
General population	15	7436	8474	-0.208	0.024	-0.255, -0.162	-8.75^∗∗^	22.93	38.939	505
Elderly	6	776	1032	-0.315	0.048	-0.409, -0.220	-6.55^∗∗^	4.265	0.000	20

*K, number of studies used for meta-analysis; NM, number of males; NF, number of females; ^∗∗^*p* < 0.01; ^∗^*p* < 0.05*.

To test whether the observed gender difference in depressive symptoms systematically varied with time, a correlation analysis between publication year and effect size of the 91 studies was carried out. This revealed no significant correlation (*r* = -0.11, *p* = 0.30). In addition, we classified all the 91 studies into two groups, those published before 2000 and those published after 2000. The 38 studies published before 2000 generated an effect size of -0.181 (*SE* = 0.032, 95% *CI* = [-0.243, -0.119], *Z* = -5.730, *p* < 0.001), with a fail-safe number of 688. The studies included were heterogeneous [*Q*(37) = 86.372, *p* < 0.01, *I*^2^ = 57.162]. Similarly, the 53 studies published in 2000 or later also generated a small but significant effect size of -0.190 (*SE* = 0.020, 95% *CI* = [-0.229, -0.151], *Z* = -9.54, *p* < 0.001), and the studies included were heterogeneous [*Q*(52) = 135.672, *p* < 0.01, *I*^2^ = 61.672.162]. The fail-safe number was 3218. The effect sizes of the two groups were not significantly different [*Q*(1) = 0.056, *p* = 0.82]. Using the 85 studies that reported the mean BDI score for each gender, correlation analyses were carried out to test whether depressive symptoms increased with publication year. No significant correlation was found (male: *r* = 0.064, *p* = 0.55; female: *r* = 0.13, *p* = 0.23. Please see **Supplementary Figure S1** for a scatter plot between depressive symptoms and year of publication for both genders).

**FIGURE 3:**
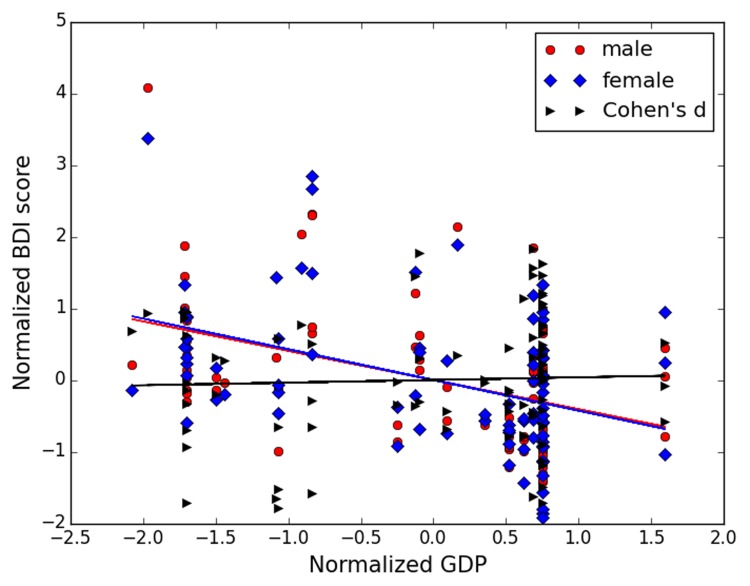
**Scatter plots between GDP (gross domestic product) per capita and male BDI (Beck Depression Inventory) score (red), between GDP per capita and female BDI score (blue), and between GDP per capita and Cohen’s *d* (black)**. All data were normalized for illustration purpose.

Next, the two measures of socioeconomic status were included as moderators for analysis. First, correlation analyses between socioeconomic indices (GDP per capita and Gini index) and BDI scores were carried out separately for both genders for the 87 studies. GDP per capita correlated negatively and significantly with BDI scores for both genders (females: *r* = -0.428, *p* < 0.01; males: *r* = -0.414, *p* < 0.01), indicating that studies carried out in areas with higher GDP per capita generated a lower mean BDI score. Similar significant correlations were observed between the Gini index and BDI scores (females: *r* = 0.336, *p* < 0.01; males: *r* = 0.356, *p* < 0.01), indicating a higher BDI score in countries/regions with a higher Gini index. To test whether gender difference in the level of depressive symptoms also varied with socioeconomic status, correlation analyses between the two socioeconomic indices and the respective effect sizes were carried out. No significant correlation was found (GDP: *r* = 0.006, *p* = 0.96; Gini index: *r* = 0.056, *p* = 0.61). **Figure 3** illustrates the relationship between the BDI score of each gender, effect sizes, and GDP. All the data were standardized.

The last moderator examined was the BDI version used. The wordings used in the BDI of a certain language may contribute to the observed gender difference in the level of depressive symptoms. As such, we separated all the studies into two groups: one group consisting of studies using an English version of the BDI and the other group consisting of studies using a non-English version of the BDI. The 47 studies using the English version of the BDI generated a small but significant effect size of -0.155 (*SE* = 0.027, 95% *CI* = [-0.208, -0.102], *Z* = -5.725, *p* < 0.01). These studies were heterogeneous [*Q*(46) = 89.701, *p* < 0.01, *I*^2^ = 48.718]. The large fail-safe-N of 710 indicated that this small effect was reliable. Similarly, the 44 studies using non-English versions of the BDI also generated a significant effect size of -0.211 (*SE* = 0.021, 95% *CI* = [-0.252, -0.170], *Z* = -10.097, *p* < 0.01). These studies were heterogeneous [*Q*(43) = 121.616, *p* < 0.01, *I*^2^ = 64.643]. The fail-safe-N was 9162. There was no significant difference between the effect sizes of the two groups [*Q*(1) = 2.754, *p* = 0.10].

In studies using the English version of the BDI, we also tested whether different versions of the BDI contributed to the observed gender difference in the level of depressive symptoms. Among the 47 studies adopting the English version of the BDI, 22 clearly stated that they used the original BDI-I (published in 1961) and 13 used the BDI-II. The 22 studies using the BDI-I generated a significant effect size of -0.172 (*SE* = 0.041, 95% *CI* = [-0.253, -0.091], *Z* = -4.149, *p* < 0.01). The studies were heterogeneous [*Q*(21) = 38.36, *p* = 0.01, *I*^2^ = 45.255] and the fail-safe-N was 142. Similarly, a small but significant effect size of -0.117 (*SE* = 0.041, 95% *CI* = [-0.198, -0.037], *Z* = -2.848, *p* < 0.01) was generated from the 13 studies using the BDI-II. These studies were heterogeneous [*Q*(12) = 22.091, *p* < 0.01, *I*^2^ = 45.679]. The fail-safe-N was 42. These two effect sizes were not significantly different [*Q*(1) = 0.865, *p* = 0.35], indicating that gender difference in the level of depressive symptoms was not likely to be influenced by the version of the BDI used.

## Discussion

The main aim of the present study was to use the meta-analytic method to ascertain whether there is a gender difference in the level of depressive symptoms in the general population. With 91 studies from five continents, we found that females did report a significantly higher level of depressive symptoms than males, with female scoring on average about 1.159 points more on the BDI than males (the highest possible BDI total score is 63). Importantly, the studies in the present meta-analysis were largely homogeneous, except those that included adolescents and young adults. This finding is interesting because most of the previous studies that did not find a gender difference in the level of depressive symptoms in non-clinical populations were based on adolescent (Teri, 1982; Martin et al., 1995; Kim et al., 2011) or university student samples (Hammen and Padesky, 1977; Gould, 1982; Bryson and Pilon, 1984; Endler et al., 1992; Steer and Clark, 1997; O’Hara et al., 1998; Pillay et al., 2002; Erol et al., 2006; Thomas and Altareb, 2012). Moreover, the gender difference in the level of depressive symptoms appeared to be independent of socioeconomic status, year of publication, and version of the BDI used. Overall, our results appear to confirm that there is a “female preponderance” in the level of depressive symptoms in the general population.

By taking into consideration two socioeconomic indices as moderators, we found a negative relationship between socioeconomic status and the level of depressive symptoms when socioeconomic status was assessed at the country/region level. Previous research on the relationship between socioeconomic status and depression was mainly carried out by assessing socioeconomic status at the individual level (Kessler et al., 2009). Taking the findings together, it can be concluded that poor socioeconomic status has a negative impact on a person not only at the individual level, but also at a country/region level. If we take depression as a continuum (Flett et al., 1997; Bowins, 2015), with healthy individuals showing mild symptoms at one end and those with a clinical diagnosis at the other end, this finding speaks against the notion that depression is a disease of modernization (Hidaka, 2012), and supports that poverty is “the pathway to depression”(McGrath et al., 1990). It is possible that in developed countries/regions, people have more knowledge about and better acceptance of mental illness, and more opportunity to have their depression diagnosed and treated. As a result, the prevalence of depression in developed countries/regions presented may be artificially inflated^3^. In contrast, in less developed countries/regions, due to poorer knowledge and acceptance of mental illness and less opportunity for diagnosis and treatment, more depressed individuals are left undiagnosed and untreated, presenting a “lower” prevalence rate.

Depression in China, where people’s acceptance of depression is not high (Xu, 1987), might offer some insight for the above point of view. The prevalence of depression in China is relatively low, with a 1-year prevalence of 1.8% and a life-time prevalence of 3.6% according to a WHO survey (Lee et al., 2009), which is much lower than the USA (Kessler et al., 2003). In another study, researchers randomly selected 50 general hospitals in Beijing in 2003–2004. A total of 73 cases who met the DSM-IV criteria for major depression were screened out among all the outpatients. Only 10 of them (14%) were identified by physicians, and only four (5%) sought help from psychologists or psychiatrists (Zhang et al., 2006). In other words, the existence of many unidentified patients may artificially “decrease” the prevalence of depression in China. This possibility is also compatible with our analysis using year of publication as a moderator. We observed that level of depressive symptoms did not vary with year of publication in the last few decades, while epidemiological studies have shown an “increasing” prevalence of depression (Joyce et al., 1990; Kessler et al., 1994; Murphy et al., 2000; Lee et al., 2007). It is possible that the increasing prevalence rate was due to an increasing number of people being diagnosed and treated secondary to increased awareness and acceptance of depression.

Another interesting finding regarding the influence of socioeconomic status on the level of depressive symptoms is that these indices had no relationship with gender difference in depressive symptoms. This suggests that women in both non-industrialized and industrialized countries/regions report a higher level of depressive symptoms than males. Previously, it has been suggested that the gender ratio of major depressive disorder in non-industrialized countries is smaller than in industrialized countries (Weissman et al., 1993). One piece of evidence that supports this viewpoint is that treated cases of depression in some non-industrialized countries indicated either equal gender ratio [e.g., Bulaways in Rhodesia (Republic of Zimbabwe after 1980) and Baghdad in Iraq; see Table 1 in Weissman and Klerman, 1977] or even a higher proportion of males than females (e.g., Dakar in Guinea, Madurai, Madras, and New Delhi in India, see Table 1 in Weissman and Klerman, 1977). Considering the economic situation as well as the level of medical service in these countries/regions, it is possible that depression in females in these countries/regions may be even more under-diagnosed than males. This issue warrants further research in these countries/regions.

The final aim of the present meta-analysis was to evaluate the validity of the three theories purporting to explain the gender difference in depression. To do this, analyses were conducted using age as a moderator. Similar to the finding previously reported in a study using patients with diagnosed depressive disorders (Angold et al., 1999), gender difference in the level of depressive symptoms was found in our youngest group, that is, the adolescents. A similar extent of gender difference in the level of depressive symptoms was also observed in young adults. The gender difference increased significantly when the group reached 30 years of age. The change in gender difference magnitude with age is consistent with the social gender role theory of depression, which is based on the disadvantaged social status that females experience relative to males (Gove, 1977; Mirowsky, 1996). In particular, it supports the age increment prediction of this theory as originally proposed by Mirowsky. Smaller gender differences are expected in adolescence since the majority of women and men would be in school and relatively few would be in paid employment. Gender difference in levels of depressive symptoms emerges in adolescence and increases significantly in middle adulthood due to the emergence of inequities in employment, housework, family responsibilities, and other factors.

The change in gender difference with age is not consistent with the biological or evolutionary theories. A biological account of gender difference, which suggests that the influence of female hormones as the cause (Janowsky and Rausch, 1985), is not consistent with the similar gender difference in the level of depressive symptoms in the middle-age and the older adult groups. We would expect female hormone levels to be different in these two periods of the human lifespan. Similarly, the evolutionary theory, which highlights females concerns about their own appearance and body image in mate selection, would predict a peak in gender difference in the level of depressive symptoms in the two younger age groups. However, our results did not support this prediction.

The embodiment of unequal social gender for adult females has been well described by those who believe that an unfavorable social gender role causes females to be at a higher risk of depression (Barnett et al., 1992; McIntosh et al., 1994). A quote from the recent Human Development Report summarized the poor situation experienced by many females worldwide: “Women experienced many kinds of disadvantage and discrimination in health, education, and employment” (United Nations Development Programme, 2014, p. 39). Thus, it appears that there is a significant link between the present finding of females scoring 1.159 points higher than males on the 21-item BDI and the observed 8% lower score of females on the Human Development Index (a comprehensive index which takes into consideration life expectancy, education, and economy) as reported by the recent Human Development Project (United Nations Development Programme, 2014). Importantly, the present findings suggest that the link may be important even at the sub-clinical level of depression in the general population.

It might be expected that the gender difference in depression would show a decline over recent decades, given improvements in education, technology, and life expectancy (United Nations Development Programme, 1990). Moreover, changes in society have begun to address the disadvantages experienced by women, particularly in industrialized countries. However, correlation analysis between year of publication and the magnitude of gender difference in the level of depressive symptoms did not support this prediction. Similar conclusions of stable gender differences in mathematical and science achievements have also been reported (Reilly et al., 2015), which further reinforces the conclusion that recent societal changes asserting women’s rights did not seem to reduce gender gaps. Such findings suggest that more specific factors may be important for the gender difference in psychological functions (Reilly and Neumann, 2013).

A limitation of the present meta-analysis is that we cannot exclude the possibility that the observed gender difference in the level of depressive symptoms is due to systematic bias in participants’ responses to certain items of the BDI. For example, several studies have shown that females are more likely to respond positively to items such as “crying” (O’Hara et al., 1998), “self-dislike,” “fatigability,” and “somatic preoccupation” (Gorenstein et al., 1999). Future studies are needed to explore and address this possibility. Additionally, the results of the present study might have been influenced by the exclusion of certain studies.

## Author Contributions

KW designed the study; HL and KW searched the literature and analyzed data; KW wrote the first version of the manuscript; other authors contributed on writing and revising the manuscript.

## Conflict of Interest Statement

The authors declare that the research was conducted in the absence of any commercial or financial relationships that could be construed as a potential conflict of interest.

## Footnotes

1 http://data.worldbank.org/indicator/NY.GDP.PCAP.CD

2 http://www.quandl.com/demography/gini-index-by-country

3 In cases of countries/regions where a hunter-gather lifestyle is dominant and contact with the “outside” world is nearly absent, there may be another explanation. However, none of the reviewed studies were conducted in such counties/regions.

## Supplementary material

FIGURE S1 **The scatter plots between mean BDI (Beck Depression Inventory) score and year of publication for female (circle) and male (square)**.
