# Supplementary figures and images for: “Female Preponderance” of Depression in Non-clinical Populations: A Meta-Analytic Study
Source: Front Psychol. 2016 Sep 15;7:1398. doi: 10.3389/fpsyg.2016.01398 (PMC5023676; doi:10.3389/fpsyg.2016.01398)

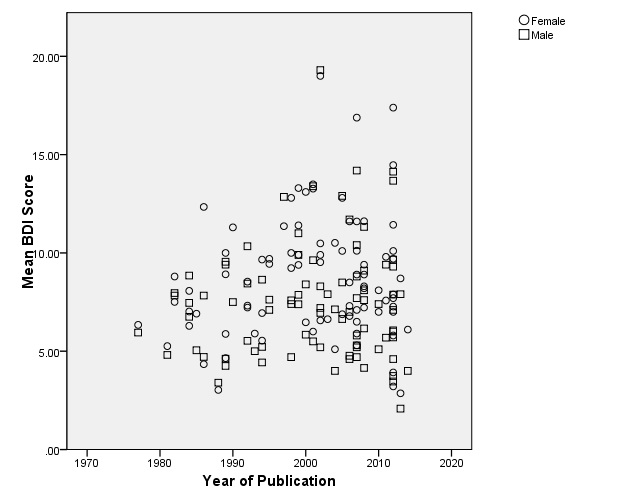

Supplement: FIGURE S1 — The scatter plots between mean BDI (Beck Depression Inventory) score and year of publication for female (circle) and male (square). [file Image_1.JPEG]
